# Spatial and Temporal Heterogeneity Analysis of Water Conservation in Beijing‐Tianjin‐Hebei Urban Agglomeration Based on the Geodetector and Spatial Elastic Coefficient Trajectory Models

**DOI:** 10.1029/2020GH000248

**Published:** 2020-08-01

**Authors:** Junhe Chen, Dongchuan Wang, Guodong Li, Zhichao Sun, Xiao Wang, Xian Zhang, Wei Zhang

**Affiliations:** ^1^ School of Geology and Geomatics Tianjin Chengjian University Tianjin China; ^2^ School of Computer and Information Engineering Tianjin Chengjian University Tianjin China

**Keywords:** InVEST model, Beijing‐Tianjin‐Hebei urban agglomeration, water conservation, geodetector model, driving factors

## Abstract

To regulate regional water resources, it is essential to identify the relationships among the elements that influence water conservation. Taking the Beijing‐Tianjin‐Hebei urban agglomeration as the study area, the authors applied a new method in combination with a geodetector model and spatial elastic coefficient trajectory model to reveal factors controlling water conservation and to identify relationships among the elements driving water conservation, in which the water conservation capacity and its spatial distribution were achieved using an Integrated Valuation of Ecosystem Services and Tradeoffs model. The authors selected precipitation, potential evapotranspiration, temperature, land use, maximum burial depth of soil, plant‐available water content, soil‐saturated hydraulic conductivity, percentage slope, gross domestic product, and population as the spatial driving factors, which measured the influence on the distribution of water conservation capacity on the whole region, plateaus, mountains, and plains, respectively. On the basis of previous research results, the authors selected precipitation, potential evapotranspiration, and land use as time‐driven factors. The results indicated that the strong water conservation capacity was reflected primarily in the Yanshan and Taihang Mountains and the eastern coastal areas. The water conservation capacity of the entire region, mountains, plateaus, and plains was affected mainly by the soil‐saturated hydraulic conductivity, plant‐available water content, precipitation, and precipitation, respectively. Each driving factor exhibited a clearly interactive influence on the spatial distribution of water conservation in terms of space and time.

## Key Points

- The authors calculated water conservation capacity of Beijing‐Tianjin‐Hebei urban agglomeration according to the InVEST model
- In terms of space, the authors used a geodetector model to explore the driving factors' interactions in relation to water conservation capacity
- In terms of time, a spatial elastic coefficient trajectory model was used to explore the interactions

## 1 Introduction

Water conservation is broadly defined, including collecting precipitation, mitigating surface runoff, inhibiting evaporation, purifying water quality, and slowing floods (Zhang et al., 2010). It is important not only for water resource planning and management and construction of hydropower stations (Kaseke & Wang, 2018; Zhang et al., 2012), but also for its key role in agriculture, aquaculture, and ecological protection. Changes in climate and land cover caused by human activities are major factors leading to changes in water conservation (Abdul‐Aziz & Ahmed, 2017; Hoyer & Chang, 2014; Miao et al., 2019). The ability to better understand the relationships among the elements driving water conservation is essential to identify trends in water conservation, is beneficial to reveal factors controlling water conservation, and is helpful to develop reasonable and effective programs for water management (Yu et al., 2015).

Since the second half of the 20th century, scholars have conducted a significant amount of research on the calculation of water conservation capacity and spatial mapping and have made several important achievements in theoretical exploration, index construction, and scale discussion (Duan et al., 2015; Gou et al., 2020; Gu et al., 2016; Miao et al., 2011, 2015; Sun et al., 2019). Because runoff generation is a complex process, many hydrological models have been established and applied to address precipitation intensity, soil permeability, slope, and vegetation, including the Stanford Watershed Model (Crawford & Linsley, 1966) and the Tank Model (Barberi et al., 2019; Hashino et al., 2002). These data require a longer research time and involve only a small part of the research field (S. Wang et al., 2008). With the development of geographic information technology, some physical hydrological models have been established, which are mainly used to simulate hydrological processes, including the Soil and Water Assessment Tool (Baker & Miller, 2013; Dennedy‐Frank et al., 2016), the System Hydrologic European tool (Refsgaard et al., 2014), and the Netherlands Hydrological Instrument (De Lange et al., 2014). These models can provide more accurate results, but the results do not reflect the spatial water‐level changes caused by changes in land use and other factors in the study area (Kuczera et al., 1993; Zheng et al., 2019). In addition, they require more data and expertise to achieve results (Vigerstol & Aukema, 2011; Zhang et al., 2014). The Integrated Valuation of Ecosystem Services and Tradeoffs (InVEST) model is a tool used for ecosystem service assessment to support environmental decision making developed in 2007 by the Stanford University, the World Wide Fund for Nature, and the Nature Conservancy (Tallis et al., 2014). The data required by the model are simple and easy to collect (Scordo et al., 2018). Compared with other hydrological models, the water yield model in InVEST has mapping and spatial analysis functions under ArcGIS. The model has been used widely in watershed water resources planning (Bai et al., 2016), hydropower and irrigation (Hamel et al., 2015), and optimal allocation of habitat resources (S. L. Liu et al., 2016).

When exploring the relationship between water conservation and its spatial driving factors, the correlation analysis method (Geng et al., 2014; Li et al., 2018; Redhead et al., 2016), random forest model (Xu et al., 2019), and Bayesian belief network (BBN) (Liao et al., 2017) are often used. Correlation analysis and the random forest model can provide importance scores for the elements driving the element set, but they are seldom used to explore the interaction characteristics among variables (S. G. Wang et al., 2018). Although a BBN describes the causal probability relationship among variables, it is difficult to update a proper BBN in an automatic and timely manner according to changes in the dependent variables process. The geodetector is a new method of spatial statistics that is independent of any linear hypothesis; the core of its theory is to detect the consistency of the spatial distribution pattern between the dependent variable and the independent variable through the spatial heterogeneity. The geodetector has a stronger power than general statistics, because it's harder for two variables to be uniformly distributed in two dimensions than it is for two variables to be uniformly distributed in one dimension. Besides, it can detect both numerical and qualitative data and can also detect the interaction of two factors on dependent variables (J. F. Wang et al., 2016). Because of its distinct advantages, it is usually applied in studies of ecology (Luo et al., 2016; Shen et al., 2015), urban transport (S. G. Wang et al., 2018), public health (Cui et al., 2019), and air pollution (Zhang & Feng, 2019). Therefore, geographic exploration is helpful to further understand the driving mechanism of water conservation.

In recent years, the ability to reveal the relationship between water conservation and its temporal driving factors according to a decoupling indicator has become an increasingly popular topic (Redhead et al., 2016). The Organization for Economic Co‐operation and Development (OECD) introduced this concept of decoupling to agricultural policy and further to ecological studies to explore the relative growth rate of various environmental factors and economic driving forces over a given period (Du et al., 2014; Tapio, 2005; Yang et al., 2010). At present, the decoupling indicator has been used widely in the field of ecology (Wang et al., 2019), especially to explore the sensitivity between driving factors and water conservation (Yu et al., 2015). Although this research has identified the relationship between water conservation and its temporal driving factors, few studies have focused on the continuous change process of the interaction relationships among temporal driving factors for water conservation (Kong et al., 2015; Xu et al., 2015). To better understand the spatiotemporal change process of water conservation, combining logic coding and traditional decoupling indicator models, the authors proposed a spatial elastic coefficient trajectory model to explore the interaction relationships among the elements driving the element set of water conservation.

To develop reasonable and effective programs for water management, the authors offered an objective framework for revealing factors to control water conservation and for identifying the relationships among the elements driving the element set of water conservation. In this study, we calculated the water yield of Beijing‐Tianjin‐Hebei urban agglomeration according to the InVEST model. On the basis of water yield results, the authors calculated water conservation with flow coefficient, soil‐saturated hydraulic conductivity, and topographic index from 2000 to 2015. The authors explored the interaction relationship between water conservation and its driving factors from the perspective of time and space. In terms of space, the authors used a geodetector model to explore the spatial heterogeneity of driving factors and their interactions in relation to water conservation capacity. The authors identified nine spatial driving factors to measure their influence on the distribution of water conservation capacity on the entire region, plateaus, mountains, and plains. In terms of time, the authors explored the temporal interaction relationship between water conservation and its driving factors according to a spatial elastic coefficient trajectory model, which the authors established by combining logical coding and decoupling indicators. The authors selected precipitation, potential evapotranspiration, and land use as the time‐driven factors.

## 2 Materials and Methods

### 2.1 Study Area

**Figure 1:**
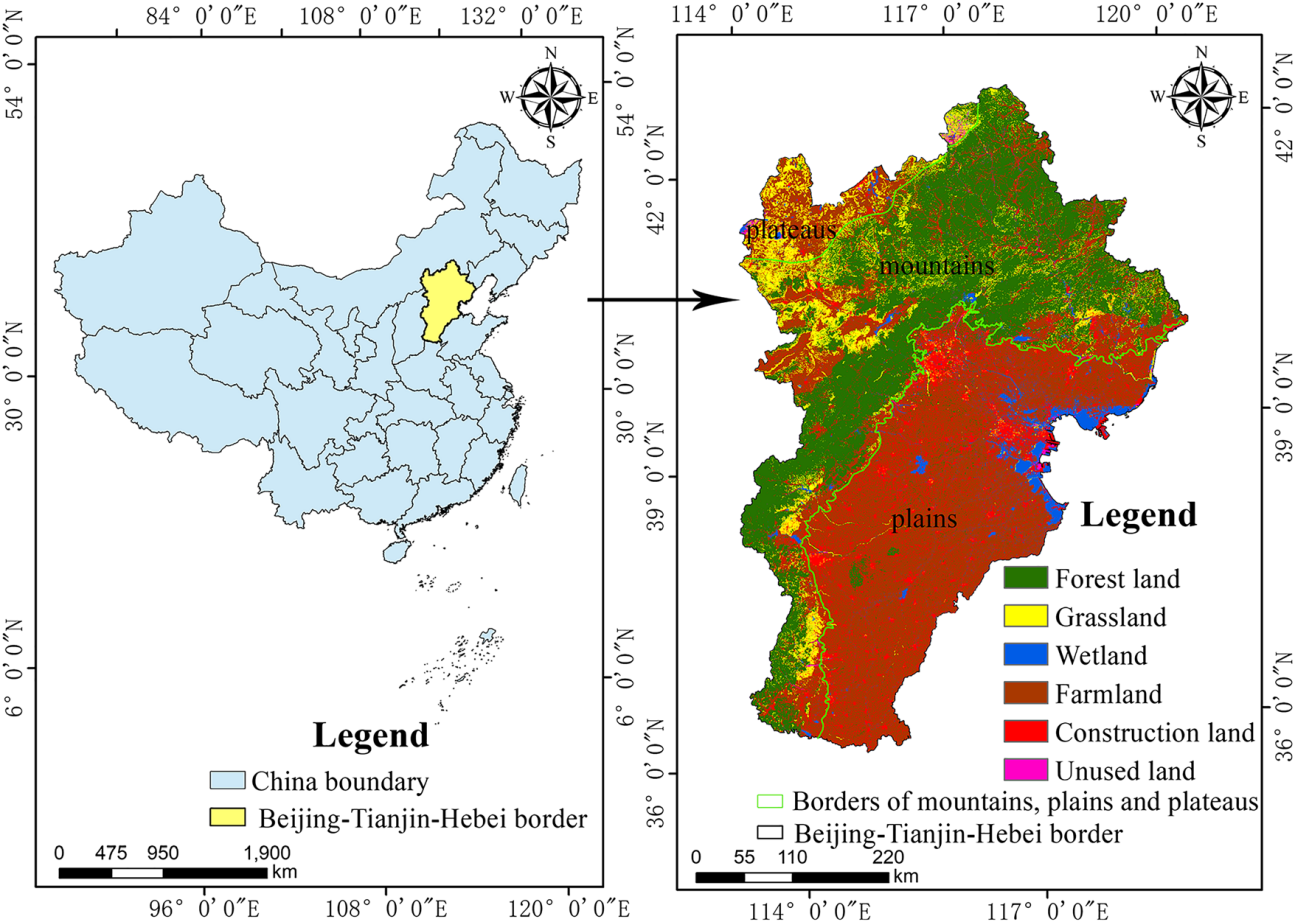
Location of the Beijing‐Tianjin‐Hebei urban agglomeration.

The Beijing‐Tianjin‐Hebei urban agglomeration is located in the eastern coastal area of China and covers a total area of 218,000 km^2^ (Figure 1). The region experiences a continental semiarid monsoon climate, which is characterized by cold‐dry winters and hot‐rainy summers. In recent years, with continued urban development, the ecological problems of the Beijing‐Tianjin‐Hebei urban agglomeration have become increasingly serious, mainly because of soil erosion, land desertification, grassland degradation, and haze. The supply of renewable water resources in the Beijing‐Tianjin‐Hebei urban agglomeration is significantly less than actual water demand, and water shortage has become an urgent problem.

Figure 1 shows the spatial extent, geographic location, and corresponding land use data of the study area.

### 2.2 InVEST Water Yield Assessment Model

The authors determine the annual water yield *Y*(*x*) of each grid unit *X* in the study area. The formula is as follows:

(1) $$Y\left(x\right)=\left(1-\frac{\mathrm{AET}\left(x\right)}{P\left(x\right)}\right)\times P\left(x\right),$$

where *P*(*x*) represents the annual precipitation (data in Chen et al., 2020) of grid cell *x* and *AET*(*x*) represents the actual evapotranspiration of grid cell *x*, where the ratio of actual evapotranspiration to precipitation is determined by the Budyko (Zhang et al., 2004) hydrothermal coupling equilibrium hypothesis:

(2) $$\frac{\mathrm{AET}\left(x\right)}{P\left(x\right)}=1+\frac{\mathrm{PET}\left(x\right)}{P\left(x\right)}-{\left[1+{\left(\frac{\mathrm{PET}\left(x\right)}{P\left(x\right)}\right)}^{w}\right]}^{\frac{1}{w}},$$

where *PET*(*x*) represents the amount of potential evapotranspiration. This formula is defined as follows:

(3) $$\mathrm{PET}\left(x\right)=K_{c}\left(l_{x}\right)\times {\mathrm{ET}}_{o}\left(x\right),$$

where *w*(*x*) represents the nonphysical parameters of natural climate‐soil properties, using the formula proposed by Donohue et al. (2012), as follows:

(4) $$w\left(x\right)=Z\frac{\mathrm{AWC}\left(x\right)}{P\left(x\right)}+1.25.$$

In the formula, *k*_*c*_(*l*_*x*_) represents the vegetation evapotranspiration coefficient of specific land use in grid unit *x*, which is used to characterize the biological characteristics of the crop and the effects of cultivation conditions on water demand and water consumption (Table 1). *Z* is the Zhang coefficient, which characterizes the annual average precipitation and other hydrogeological characteristics. *AWC*(*x*) indicates the soil's effective water content, determined by the plant's water use content (PAWC), and the maximum root burial depth of the soil and the root depth of the plant. The maximum root burial depth of the soil refers to the maximum root depth of the vegetation cover and the maximum depth that the plant roots can extend in the soil. Plant root depth usually refers to the soil depth of 95% of the root biomass of a particular plant type, which is assigned according to the InVEST model parameter table (Table 1), which was based on the research results of Fu et al. (2013) and Bao et al. (2016). PAWC uses the calculation method of Zhou et al. (2005), as follows:

(5) $$\text{PAWC}=54.509-0.132\times \text{sand}\%-0.003\times {\left(\text{sand}\%\right)}^{2}-0.055\times \text{silt}\%-0.006\times {\left(\text{silt}\%\right)}^{2}-0.738\times \text{clay}\%+0.007\times {\left(\text{clay}\%\right)}^{2}-2.688\times \mathrm{om}\%+0.501\times {\left(\mathrm{om}\%\right)}^{2},\text{and}$$

(6) $$\mathrm{AWC}\left(x\right)=\mathrm{Min}\left(\text{Rest}.\text{layer}.\text{depth},\text{root}.\text{depth}\right)\times \text{PAWC}.$$

**Table 1:** Parameters for InVEST Model

Land use	The vegetation evapotranspiration coefficient	Root depth (mm)	Flow velocity coefficient
Forestland	1.2	7,000	300
Grassland	0.65	2,600	500
Wetland	1	1,000	2,012
Cultivated land	0.8	2,100	400
Artificial surface	0.275	500	800
Other land	0.2	255	1,500
Sea area	1	1,000	2,012

In the formula, sand%, silt%, clay%, and om% are the percentages of sand, powder, clay, and soil organic matter in soil texture, respectively. *ET*_*o*_(*x*) represents the reference crop evapotranspiration (data in Chen et al., 2020) of grid cell *x*, and the reference crop evapotranspiration is calculated using the Hargreaves method (Hargreaves & Samani, 1985):

(7) $${\mathrm{ET}}_{o}\left(x\right)=0.0023\times \frac{R_{a}}{\lambda }\times \sqrt{T_{x}-T_{n}}\times \left(T+17.8\right),$$

where *Ra* is the atmospheric top‐layer radiation [*MJ*/(*m* ^2^*d)], which can be calculated from latitude (Zhang et al., 2012); λ represents the latent heat of vaporization, λ = 2.45 MJ/kg (Allen et al., 1998); *T*_*x*_ and *T*_*n*_ are the daily average maximum temperature and the daily average minimum temperature, respectively; and *T* is the average of the daily maximum temperature and the daily minimum temperature.

The vegetation evapotranspiration coefficient, root depth, and flow velocity coefficient of each land use type are shown in Table 1.

### 2.3 Water Conservation Calculation Method

The authors used water yield based on the InVEST model, combined with flow coefficient, soil‐saturated hydraulic conductivity, and topographic index to calculate water conservation (Nelson et al., 2009):

(8) $$\text{Retention}=\min\left(1,\frac{249}{\text{velocity}}\right)\times \min\left(1,\frac{0.9\times \mathrm{TI}}{3}\right)\times \min\left(1,\frac{k_{\mathrm{sat}}}{300}\right)\times Y\left(x\right),$$

where retention is the water conservation (mm), velocity is the flow velocity coefficient (Table 1), and *k*_*sat*_ is the soil‐saturated hydraulic conductivity (mm/d). This refers to the amount of water flowing through the unit area per unit time under the unit water potential gradient when the soil is saturated with water. It is calculated according to the Cosby model (Cosby et al., 1984), where *Y(x)* is the yield (mm) and *TI* is the topographic index, dimensionless, as calculated by formula 9:

(9) $$\mathrm{TI}=\mathrm{lg}\left(\frac{\text{drainage}\_\text{area}}{\text{soil}\_\text{depth}\times \text{percent}\_\text{slope}}\right),$$

where drainage_area is the number of grids in the catchment area, dimensionless; soil_depth is the soil depth (mm); and percent_slope is the percentage slope, based on the digital elevation model (DEM) data with a spatial resolution of 30 m, calculated using the global information system (GIS) three‐dimensional (3D) analysis tool.

### 2.4 Geodetector Model

The authors used the geodetector model, a new spatial analysis method, to assess the relationship between driving factors and relevant resultant outcomes. There are many available geodetector models. In this study, the authors focused on two: factor detection and interactive detection (J. F. Wang et al., 2016).

The factor detector, which is a type of geodetector, can test whether a particular factor is the reason for certain special distribution diversity. It does so by comparing the total variance of the index in a subregion with that in the entire region:

(10) $$\;P_{D,H}=1-\frac{1}{n\sigma_{H}^{2}}\sum_{i=1}^{m}n_{D,i}\sigma_{H_{D,i}}^{2},$$

where *D* is the influencing factor; *H* is the affected index; *P*_*D*,*H*_ is the effect of *D* on *H*; *n* and *σ*^2^ are the number and variance of the samples, respectively; *m* is the classification number of an index; and *n*_*D*,*i*_ is the sample number of *D* of type *i*. Within the range of values from 0 to 1, the larger *P*_*D*,*H*_ is, the more it can influence a region.

The interactive detector, which is an advancement in geodetectors over other statistical methods, compares the *q* values of single factors and of interactive factors. It is used to explore whether the two factors, when taken together, weaken or enhance one another, or whether they have completely independent effects on a research subject (Ran et al., 2019; J. F. Wang et al., 2016).

The judgment method of the interaction mode is shown in Table 2.

**Table 2:** Types of Interaction Between Two Covariates

Interaction	Judgment criteria
Enhance	q(X1∩X2) > q(X1) or q(X2)
Enhance, bivariate	q(X1∩X2) > q(X1) and q(X2)
Enhance, nonlinear	q(X1∩X2) > q(X1) + q(X2)
Weaken	q(X1∩X2) < q(X1) + q(X2)
Weaken, univariate	q(X1∩X2) < q(X1) or q(X2)
Weaken, nonlinear	q(X1∩X2) < q(X1) and q(X2)
Independent	q(X1∩X2) = q(X1) + q(X2)

*Note*. X1 and X2 represent the driving factors of water conservation. The symbol ∩ denotes the interaction between X1 and X2.

### 2.5 Spatial Elastic Coefficient Trajectory Model

The spatial elastic coefficient trajectory model is used to explore whether two factors, when taken together, weaken or enhance one another, or whether they have completely independent effects on a research subject from time.

The authors used the decoupling indicator introduced by the OECD (Du et al., 2014; Yu et al., 2015) to describe the relationship between water conservation and temporal driving factors, as follows:

(11) $$\eta_{i}=\frac{u_{\mathrm{wy}}}{u_{p}}=\frac{\left({\mathrm{WY}}_{t}-{\mathrm{WY}}_{t_{0}}\right)/{\mathrm{WY}}_{t0}}{\left(P_{t}-P_{t_{0}}\right)/P_{t_{0}}},$$

where *η*_*i*_ is the elasticity coefficient of water conservation and temporal driving factors during the *i*th year (*i* = 2000, 2015); *WY* and *WY*_*t*0_ are water conservation from *t*_0_ to *t* years (*t*_0_ to *t* are years 2000–2015); *P*_*t*_ and $P_{t_{0}}$ are temporal driving factors from *t*_0_ to *t* years; and *u*_*wy*_ and *u*_*p*_ are change rate of water conservation and temporal driving factors, respectively.

The authors calculated the elasticity coefficient of the temporal driving factors and water conservation from 2000 to 2015 based on the decoupling model. On this basis, and combining logic code, the authors built a space elastic coefficient trajectory of water conservation capacity with precipitation, potential evapotranspiration, and land use to describe the sensitivity of the water conservation capacity to precipitation, potential evapotranspiration, and land use, respectively. Equation 3 provided the spatial elastic coefficient trajectory model:

(12) $$T_{\mathrm{ij}}={\left(G1\right)}_{\mathrm{ij}}\times 10^{n-1}+{\left(G2\right)}_{\mathrm{ij}}\times 10^{n-2}+\ldots +{\left(\mathrm{Gn}\right)}_{\mathrm{ij}}\times 10^{n-n},$$

where *T*_*ij*_ is the logical coding value of column *j* and row *i* in the raster image of logical analysis results, which indicates the value of elastic coefficient of different grades. The values of the elastic coefficients of different grades are represented by 1, 2, 3, …, where the higher the grade, the larger the elastic coefficient value, and *n* is the number of temporal driving factors. The present study looked at three temporal factors. Therefore, *n* = 3 and *G*1_*ij*_, *G*2_*ij*_, and *G*3_*ij*_ are the logical coding of the corresponding raster types on the raster images of precipitation, potential evapotranspiration, and land use, respectively, from 2000 to 2015.

According to reclassification and the grid calculation method, we obtained the spatial elastic coefficient trajectory code. By referring to the classification results of Yu et al. (2015) and combined with the natural environment characteristics of the study area, we divided the calculated elastic coefficient results into three grades from low to high (1, 2, and 3)—the higher the coefficient of elasticity, the worse the ability to regulate the change of precipitation, potential evapotranspiration, and land use, and the more sensitive these areas were. Therefore, the authors divided the study area into six parts: strong precipitation regulation area, strong potential evapotranspiration regulation area, strong land use regulation area, strong comprehensive (precipitation, potential evapotranspiration, and land use) area, weak regulation area, and no data. Among these, strong precipitation regulation indicated that the elastic coefficient of precipitation and water conservation was 1, and strong comprehensive regulation indicated that the elastic coefficient of two or three temporal driving factors and water conservation was 1. For example, if the logical coding was “123,” it meant that the elastic coefficients of precipitation, potential evapotranspiration, and land use were 1, 2, and 3, respectively. Because the elastic coefficient of precipitation and water conservation was 1, “123” corresponded to the strong precipitation regulation area. Besides, if the proportion of the strong comprehensive regulation area is greater than the strong precipitation (potential evapotranspiration/land use) regulation area, it indicates that the influence of multiple factors on water conservation in the study area is greater than that of single factor; otherwise, it indicates that the single factor in the study area has a stronger ability to regulate water conservation.

### 2.6 Selection of Influencing Factors of Water Conservation

**Figure 2:**
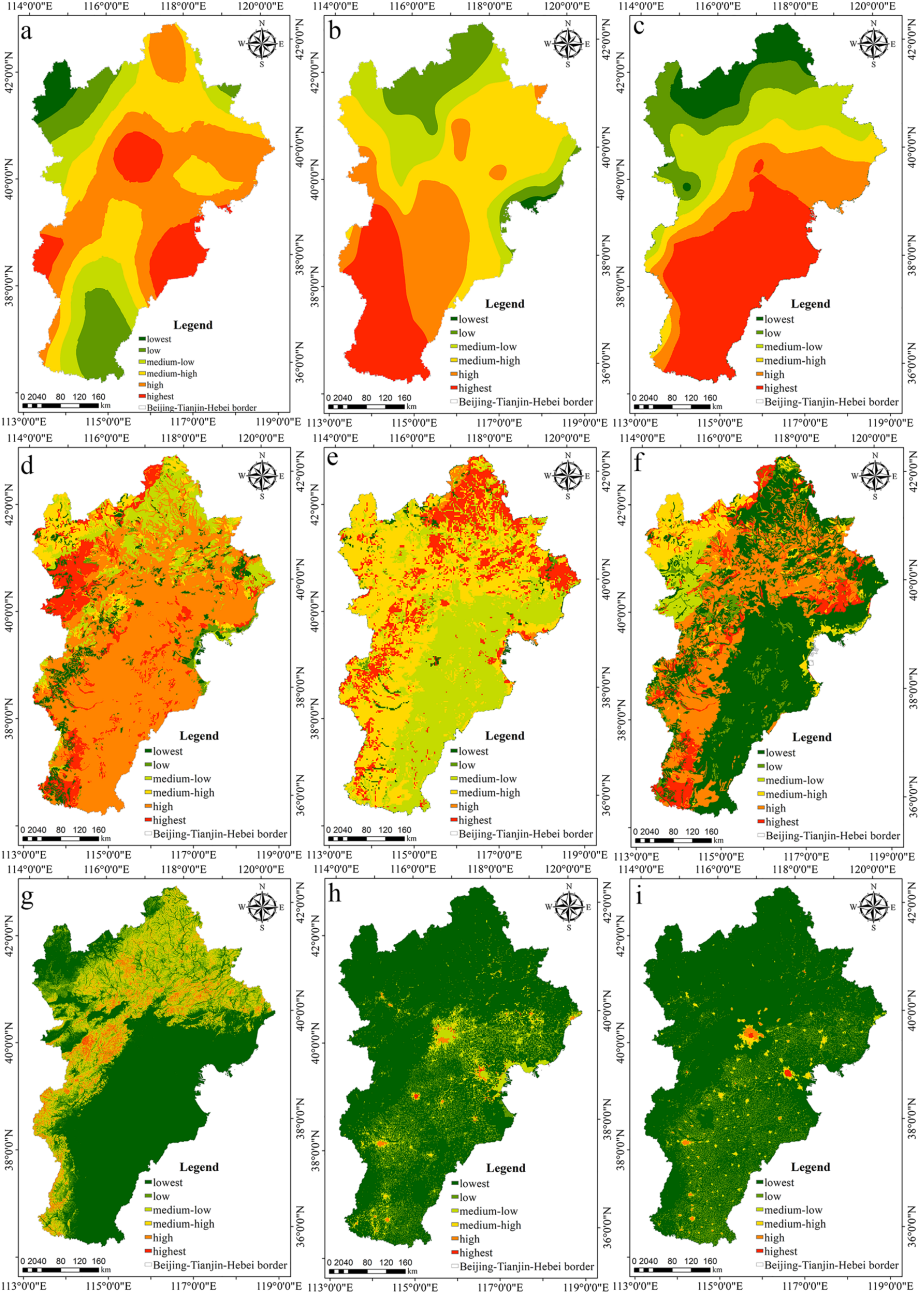
Spatial patterns of spatial driving factors: (a) precipitation, (b) potential evapotranspiration, (c) temperature, (d) the maximum buried depth of soil roots, (e) plant‐available water content, (f) soil‐saturated hydraulic conductivity, (g) percentage slope, (h) GDP, and (i) population in 2015.

The selection principle of the influencing factors mainly considers regional integrity, spatial heterogeneity, and the interrelation of the influencing factors. That is to say, the impact factors must cover the entire region, and the spatial distribution is different. Also referring to the research results of multiple scholars (Lang et al., 2017; Zeng & Li, 2019), the authors selected the driving factors of water conservation from the perspectives of society and nature, including precipitation, potential evapotranspiration, temperature, land use, maximum burial depth of soil, plant‐available water content, soil‐saturated hydraulic conductivity, percentage slope, gross domestic product (GDP), and population (Figure 2). Among them, maximum burial depth of soil, plant‐available water content, and soil‐saturated hydraulic conductivity were obtained based on soil data; percentage slope was obtained based on DEM data; temperature data were mainly used for the calculation of potential evapotranspiration; and GDP and population were not involved in the calculation of water conservation. Therefore, these factors had little impact on water conservation. Referring to the research results of scholars (Yu et al., 2015; Zhang et al., 2012) at the same time, the authors tested the input data several times and found that precipitation, potential evapotranspiration, and land use had a greater impact on water conservation at the time level. Therefore, the authors calculated the water conservation capacity from 2000 to 2015 under the condition that other input data did not change, while the three focal data points changed across a given year. The study period of spatial heterogeneity of water conservation was 2015, and the study period of temporal heterogeneity of water conservation was from 2000 to 2015.

Based on the remote sensing images, DEM data and social data of the study area (data in Chen et al., 2020), precipitation, potential evapotranspiration, temperature, the maximum buried depth of soil roots, plant‐available water content, soil‐saturated hydraulic conductivity, percentage slope, GDP, and population are calculated and shown in Figure 2.

## 3 Data

The authors obtained the data used in this paper mainly from the following sources: meteorological data (2000–2015) (China Meteorological Data Network, 2000–2015), Landsat satellite images (2000–2015) (NASA's Earth Observing System Data and Information System, 2000–2015), China City Statistics Yearbook (2015) (National Bureau of Statistics, 2015), soil data (the Soil Science Data Center of the National Earth System Science Data Sharing Service Platform), and DEM (Geospatial Data Cloud).

## 4 Results

### 4.1 Analysis of Spatial and Temporal Patterns of Water Conservation Capacity

**Figure 3:**
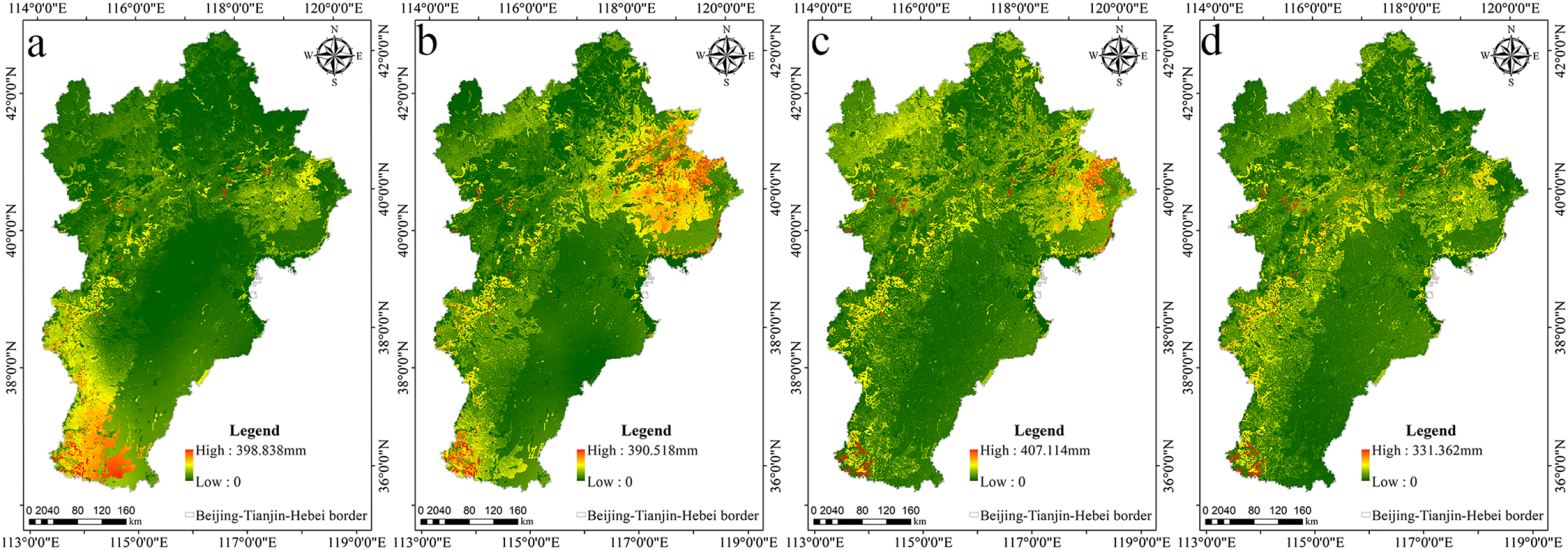
Spatial patterns of water conservation: (a) in 2000, (b) in 2005, (c) in 2010, and (d) in 2015.

In 2000, the high‐water conservation area of the Beijing‐Tianjin‐Hebei urban agglomeration was distributed primarily in Handan, for which average water conservation capacity was 86.341 mm (Figure 3a; Table 3). In 2005, the high‐water conservation area of the Beijing‐Tianjin‐Hebei urban agglomeration was distributed primarily in the eastern coastal areas. The water conservation capacity of Tangshan was the largest, at 56.057 mm, and the water conservation capacity in the central and southern part of the Beijing‐Tianjin‐Hebei urban agglomeration was relatively low (Figure 3b; Table 3). In 2010, the high‐water conservation area of the Beijing‐Tianjin‐Hebei urban agglomeration was basically the same as that in 2005, and the average water conservation of Qinhuangdao and Tangshan was relatively large, at 46.969 and 42.089 mm, respectively (Figure 3c; Table 3). In 2015, the high‐water conservation area of the Beijing‐Tianjin‐Hebei urban agglomeration was distributed primarily in the Yanshan and Taihang Mountains, and the water conservation capacity in the northwestern area and the southern plain area was relatively less than that in other parts of the study area (Figure 3d; Table 3). On the whole, a strong water conservation capacity was reflected primarily in the Yanshan and Taihang Mountains and the eastern coastal areas, which were rich in precipitation, had developed surface water systems, or had good vegetation coverage.

**Table 3:** Average Water Conservation Capacity of the Beijing‐Tianjin‐Hebei Urban Agglomeration From 2000 to 2015

City	Average water conservation capacity in 2000 (mm)	Average water conservation capacity in 2005 (mm)	Average water conservation capacity in 2010 (mm)	Average water conservation capacity in 2015 (mm)
Beijing	14.838	21.553	21.851	29.402
Qinhuangdao	18.899	39.615	46.969	19.168
Chengde	7.923	28.635	22.594	16.130
Zhangjiakou	15.258	13.993	29.469	21.578
Baoding	12.272	14.802	11.840	22.516
Tianjin	7.595	15.964	12.870	16.381
Tangshan	27.611	56.057	42.089	29.731
Langfang	3.958	12.640	10.859	18.652
Shijiazhuang	37.613	18.004	11.487	24.411
Canzhou	13.172	9.526	12.372	17.914
Hengshui	17.826	7.846	7.549	10.725
Xingtai	53.573	17.137	10.661	12.530
Handan	86.341	42.127	23.748	20.218

Average water conservation capacity of the Beijing‐Tianjin‐Hebei urban agglomeration from 2000 to 2015 is shown in Table 3.

Based on the InVEST model, the spatial patterns of water conservation from 2000 to 2015 are calculated and shown in Figure 3.

### 4.2 Analysis of Geodetector Results

#### 4.2.1 Analysis of Factor Detection Results

The authors took water conservation as dependent variable and driving factor as independent variable. Because the independent and dependent variables were spatial data, in order to match these two variables in space, the grid method was used to achieve the corresponding variable values. If the density of grid points was large, the accuracy of the calculation result will be high, but the calculation amount will also be large. Therefore, it was necessary to consider the balance between accuracy and efficiency in actual operation. Since the authors resampled all data to spatial resolution of 1,000 m, in order to ensure more accurate results, the authors repeatedly tested the research data and selected 1 km as the grid scale. The authors measured the influence of the driving factors on the distribution of water conservation capacity by factor detection—that is, the larger the *q* value, the greater the impact of the driving factors on water conservation. The order was as follows (the number in parentheses was the corresponding *q* value): soil‐saturated hydraulic conductivity (27.141%) > precipitation (18.722%) > plant‐available water content (12.642%) > temperature (12.583%) > potential evapotranspiration (9.885%) > maximum root burial depth of soil (5.332%) > land use (3.575%) > percentage slope (2.485%) > GDP (1.534%) > population (1.423%). The specific explanation was that the soil‐saturated hydraulic conductivity, precipitation, and plant‐available water content had a higher explanatory power for water conservation than the other factors. In Yanshan and Taihang Mountains, water conservation capacity was relatively strong (Figure 3d); there was not only abundant precipitation and plant‐available water content, but also strong soil‐saturated hydraulic conductivity (Figures 2a, 2e, and 2f). In the plain region, water conservation capacity was relatively weak, due to the relatively high value of GDP and population (Figures 2h and 2i).

#### 4.2.2 Analysis of Interactive Detection Results

The authors used interactive detection to determine whether interaction relationships existed among different factors influencing the spatial distribution of water conservation. The interactive detection results indicated that a spatial interaction did exist between the driving factors. After any two of the driving factors interacted, the factors influenced were either two‐factor enhancement or nonlinear enhancement. Among these enhancements, the influence between precipitation and soil‐saturated hydraulic conductivity had the highest value at 0.547 (Table 4). The effects of the two‐factor interactions on water conservation capacity were greater than the effects of any individual factor. Uneven distribution of water conservation was the result of a combination of factors.

**Table 4:** Interactive Detection Results

	Precipitation	Potential evapotranspiration	Temperature	Land use	Maximum root burial depth of soil	Plant‐available water content	Soil‐saturated hydraulic conductivity	Percentage slope	GDP	Population
Precipitation	0.187@									
Potential evapotranspiration	0.257*	0.099@								
Temperature	0.309*	0.197*	0.126@							
Land use	0.270#	0.181#	0.228#	0.036@						
Maximum root burial depth of soil	0.367#	0.193#	0.223#	0.113#	0.053@					
Plant‐available water content	0.407#	0.287#	0.308#	0.181#	0.230#	0.126@				
Soil‐saturated hydraulic conductivity	0.547#	0.398#	0.353*	0.304*	0.370#	0.390*	0.271@			
Percentage slope	0.225#	0.139#	0.207#	0.065#	0.106#	0.184#	0.301#	0.025		
GDP	0.224#	0.149#	0.178#	0.054#	0.076#	0.145#	0.300#	0.048#	0.015@	
Population	0.222#	0.141#	0.176#	0.049*	0.071#	0.144#	0.291#	0.044#	0.027*	0.014@

*Note*. Add @ as the factor detection result, add * interaction to two‐factor enhancement, plus # is nonlinear enhancement.

The effects of the two‐factor interactions on water conservation capacity are shown in Table 4.

### 4.3 Analysis of Spatial Elastic Coefficient Trajectory

**Figure 4:**
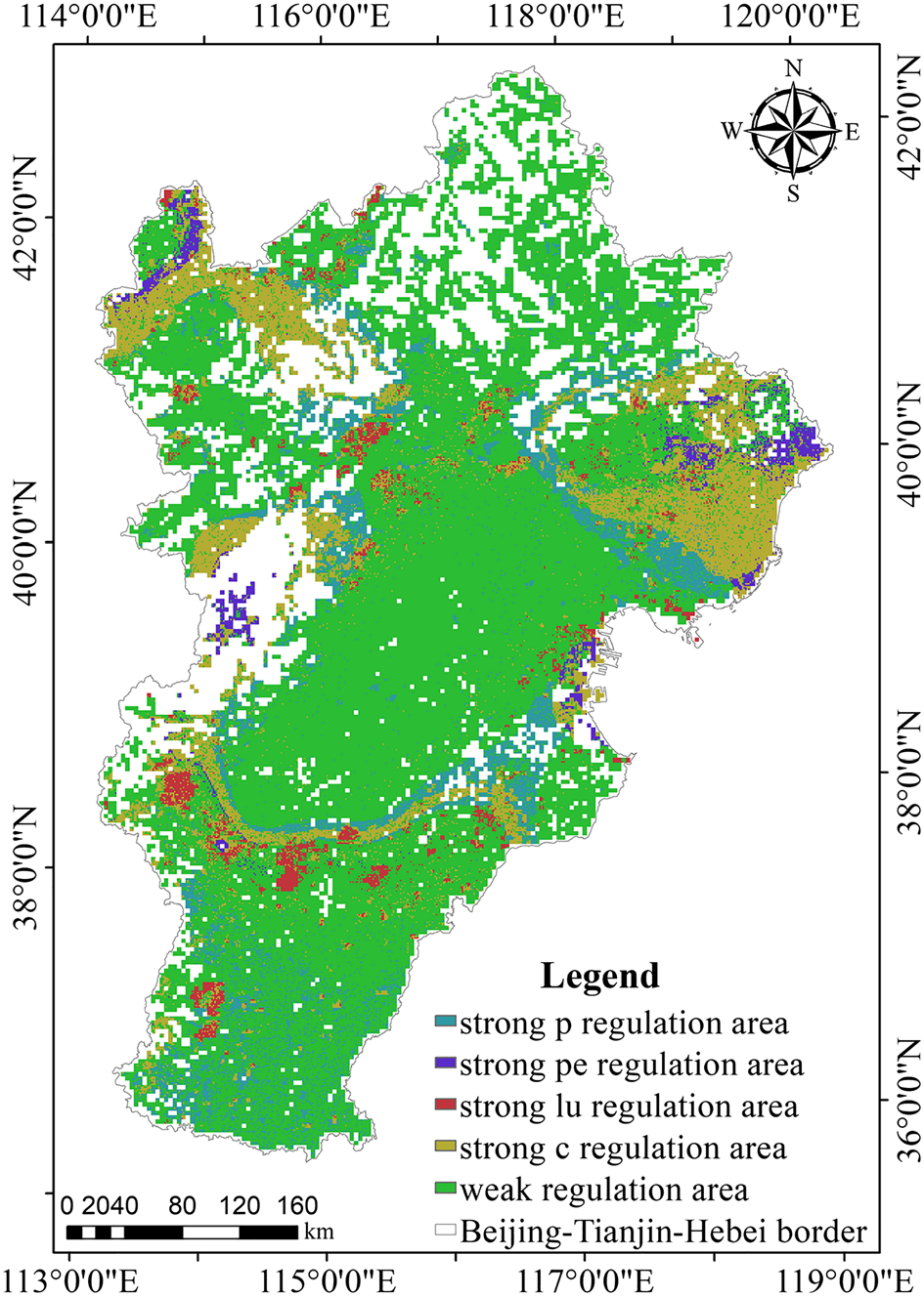
Spatial elastic coefficient trajectory map.

Since precipitation and potential evapotranspiration were numerical quantities, they can be directly involved in the calculation of the formula, while land use was the type quantity. The authors used the variation of the area of each land use type to represent the impact of land use on water conservation. The units of precipitation, potential evapotranspiration, and land use are not uniform, and the authors dealt with them by min‐max normalization (Munkhdalai et al., 2019). According to reclassification and the grid calculation method, we obtained a spatial elastic coefficient trajectory table (Table 5) and map (Figure 4). The study area had 27 codes, which accounted for 76.822% of the total area (the white part in the figure was “no data”). When calculating the elastic coefficient of land use and water conservation, we used the grid method to calculate the rate of change in the land use area. Because of the rapid development of urbanization, a large number of land use types were transformed into construction land, and although forestland changes were small, forestland was distributed primarily in these null parts. As a result, the authors labeled this area as infinity, which was not analyzed in this paper.

**Table 5:** Spatial Elastic Coefficient Trajectory Table

Regulation area	Code	Proportion (%)
Strong p regulation area	122,123,132,133,	7.924
Strong pe regulation area	212,213,312,313,	1.688
Strong lu regulation area	221,321,331,231	2.303
Strong c regulation area	111,112,113,121,131,211,311	11.134
Weak regulation area	222,223,232,233,322,323,332,333	53.773
No data	—	23.178

*Note*. p, precipitation; pe, potential evapotranspiration; lu, land use; and c, comprehensive.

The corresponding codes and proportions of each regulation area are shown in Table 5.

The spatial elastic coefficient trajectory map was retrieved using the spatial elastic coefficient trajectory model and GIS analysis (Figure 4).

Compared with the regulation area of strong potential evapotranspiration and strong land use, the regulation area of strong precipitation was relatively large. This showed that the sensitivity between precipitation and water conservation was relatively low, and the study area had the best effect on precipitation regulation. On the whole, the low‐sensitivity area occupied 23.049% of the study area, which was distributed mainly in the northwest plateau area, the Yanshan and Taihang Mountains, and the eastern coastal area. These regions held a stronger self‐regulation capacity in precipitation, potential evapotranspiration, and land use, to some extent, which proved the stability of water conservation capacity in these areas. The high‐sensitivity area occupied 53.773% of the study area, which indicated that water conservation capacity was sensitive to changes in precipitation, potential evapotranspiration, and land use in the entire region. The proportion of the strong comprehensive regulation area is greater than the strong precipitation (potential evapotranspiration/land use) regulation area, which indicated that the combined effect of precipitation, potential evapotranspiration, and land use on water conservation was larger than any single factor of three. Therefore, when formulating reasonable policies to regulate regional water resources, the government should comprehensively consider the common influence of various factors, and these factors should be adapted to local conditions for the maintenance of regional surface water resource allocation.

## 5 Discussion

### 5.1 Comparison of Water Conservation Results

On the basis of changes in precipitation, potential evapotranspiration, and land use in 2000, 2005, 2010, and 2015, we simulated the water yield for 2000, 2005, 2010, and 2015. To verify the water yield simulated by the InVEST model, according to the total water resources for 2000, 2005, 2010, and 2015 provided by the “Water Resources Bulletin of Hebei Province,” “Beijing Water Resources Bulletin,” and “Tianjin Water Resources Bulletin,” we calculated the actual water yield of Beijing‐Tianjin‐Hebei urban agglomeration in each year. The authors adjusted the Zhang coefficient for actual water yield for each year and simulated water yield several times. The authors controlled the relative error of water yield in each year within ±0.03. When the Zhang coefficients for 2000, 2005, 2010, and 2015 were 4.5, 4, 8.2, and 5, respectively, the water yield was well simulated, with a water yield of 16.461, 166.88, 17.424, and 17.567 billion cubic meters (Table 6), which showed an increasing trend.

**Table 6:** Water Yield Calibration Table, 2000–2015

Year	2000	2005	2010	2015
Actual water yield (100 million cubic meters)	164.38	168.38	170.09	174.67
InVEST model water yield (100 million cubic meters)	164.61	166.88	174.24	175.67
Relative error	0.001	0.009	−0.024	−0.006
Zhang coefficient	4.3	4	6.2	5.5

Actual water yield, InVEST model water yield, relative error, and Zhang coefficient of the study area from 2000 to 2015 are shown in Table 6.

**Figure 5:**
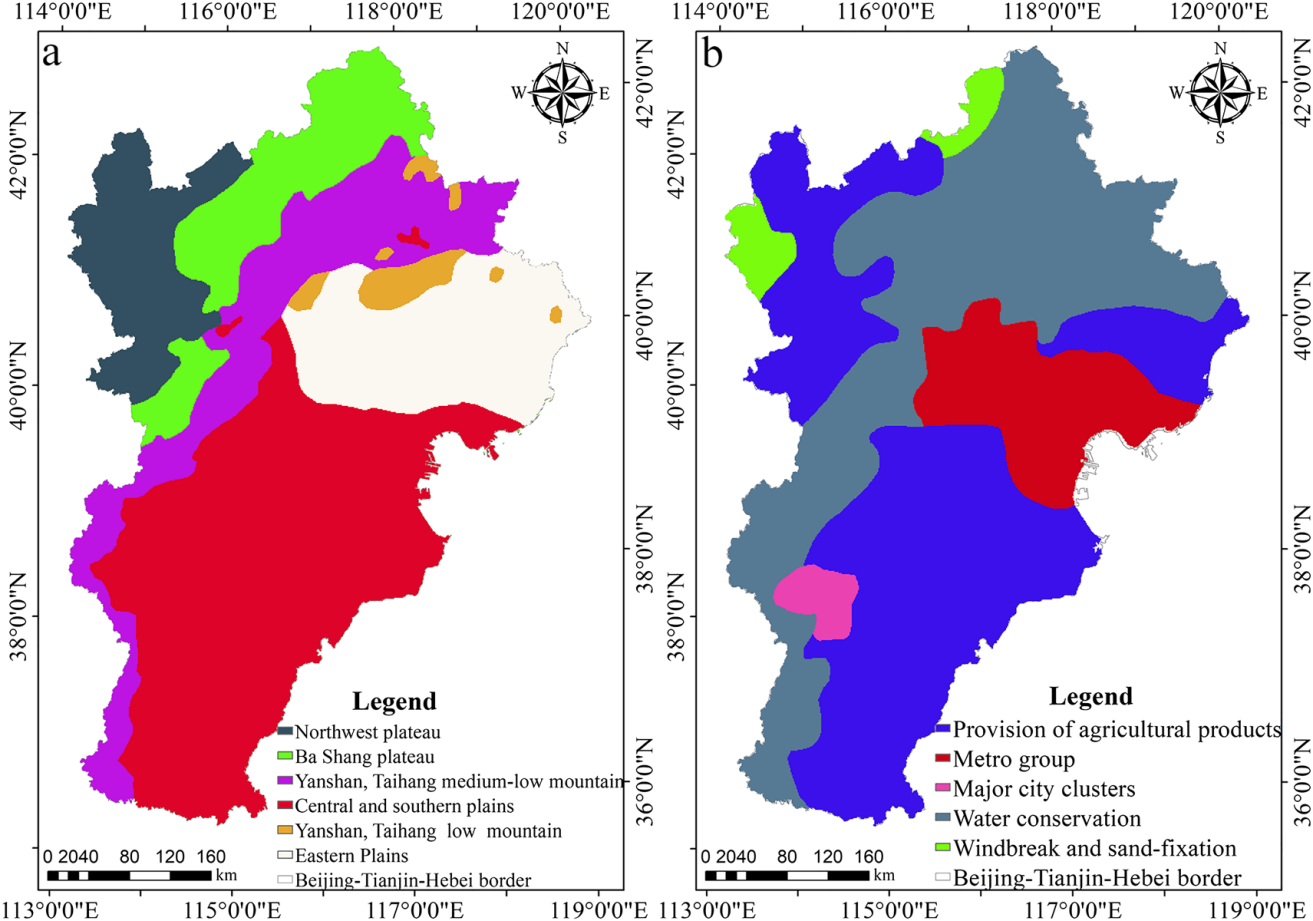
Comparison of spatial patterns of water conservation: (a) SOFM neural network partition and (b) the national ecological functional zoning.

Y. Liu et al. (2015) used the self‐organizing feature map (SOFM) neural network to divide the Beijing‐Tianjin‐Hebei region into six water conservation functions based on the influence of such factors as altitude, precipitation, evapotranspiration, soil‐saturated water content, and forest coverage (Figure 5a). The Ministry of Environmental Protection and the national ecological functional zoning, supported by the Chinese Academy of Sciences, divided the Beijing‐Tianjin‐Hebei Ecological Function Zone into five sections in 2015 (Figure 5b). This paper calculated the average water conservation capacity of the Beijing‐Tianjin‐Hebei urban agglomeration from 2000 to 2015 through GIS analysis and calculated the average water conservation capacity of the SOFM neural network partition and the national ecological functional zoning in each area, respectively. The results indicated that the average water conservation capacity in Yanshan, Taihang medium‐low mountain area; Yanshan, Taihang low mountain area; and the eastern plains was greater than that in other regions (Figure 5a; Table 7). The average water conservation capacity in the water conservation area, the metropolitan group, and major city clusters was greater than that in other regions (Figure 5b; Table 7). These results indicated that the strong water conservation capacity in the study area was distributed primarily in Yanshan, Taihang, and eastern coastal areas, which was consistent with the conclusions of this study.

**Table 7:** Statistical Table of Water Conservation in SOFM Neural Network Subarea

SOFM neural network partition	Average water conservation (mm)
Northwest plateau	22.538
Ba Shang plateau	19.270
Yanshan, Taihang medium‐low mountain area	30.494
Central and southern plains	22.648
Yanshan, Taihang low mountain area	24.111
Eastern plain	38.820

The spatial pattern of SOFM neural network partition is interpreted by referring to the result of Y. Liu et al. (2015) (Figure 5a); the spatial pattern of the national ecological functional zoning is interpreted by referring to the Beijing‐Tianjin‐Hebei Ecological Function Zone (Figure 5b).

Based on the average water conservation of the paper, the SOFM neural network partition's average water conservation are calculated and shown in Table 7.

Based on the average water conservation of the paper, the national ecological function zone's average water conservation are calculated and shown in Table 8.

**Table 8:** Statistical Table of Water Conservation in National Ecological Function Regionalization

National ecological function zone	Average water conservation (mm)
Water conservation	30.816
Winbrea and sand fixation	21.397
Provision of agricultural products	23.056
Metropolitan group	23.569
Major city clusters	30.889

### 5.2 Discussion of Geodetector Results

**Figure 6:**
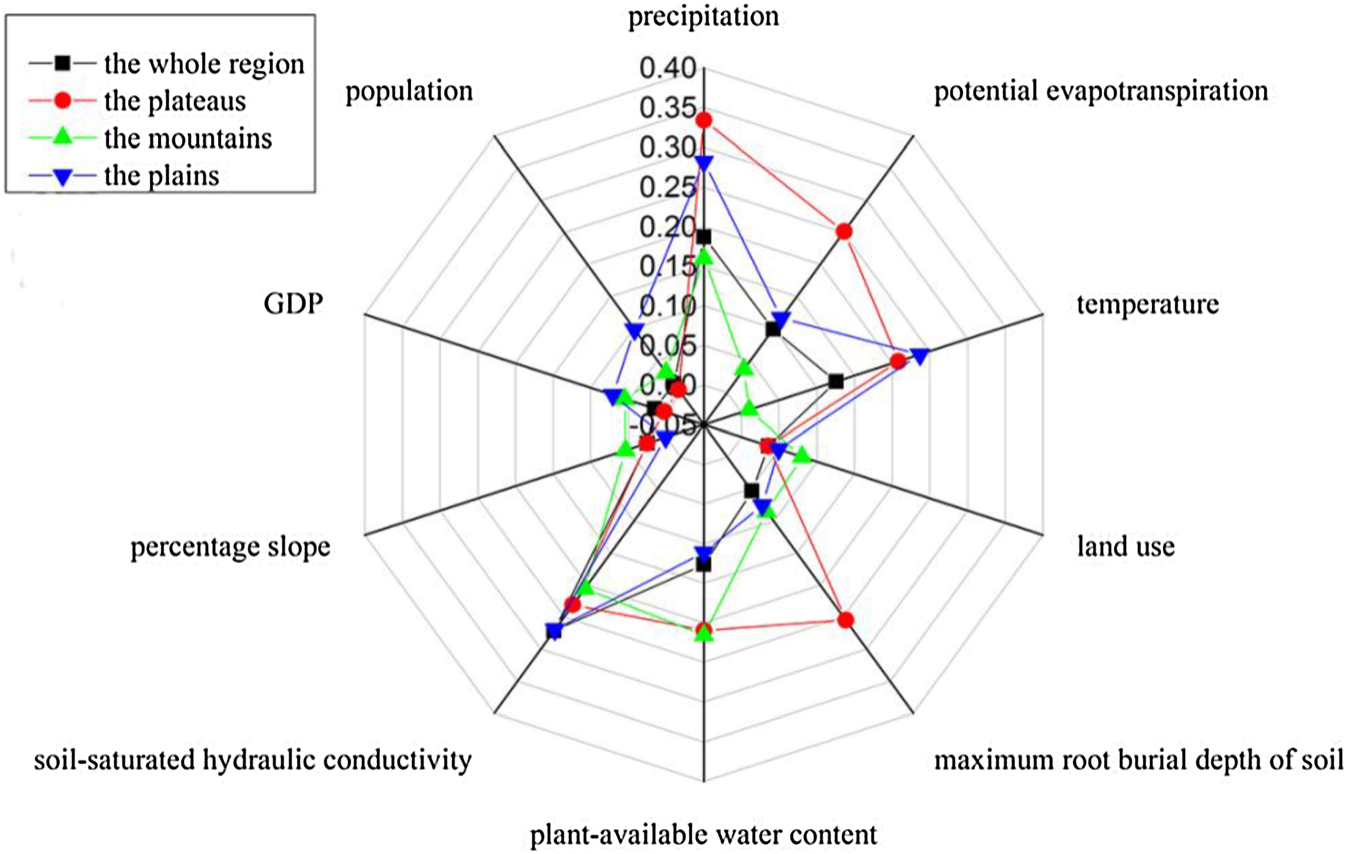
The *q* values of the driving factors for water conservation on (a) the whole region, (b) the plateaus, (c) the mountains, and (d) the plains.

Understanding the driving mechanism of water conservation in the Beijing‐Tianjin‐Hebei urban agglomeration could provide a reference for establishing a regional water resources planning strategy and could guide the planning mode of water resources according to local conditions. The authors found that water conservation was higher in Yanshan and Taihang Mountains, but less in the plain area and plateau area of central and southern of the study area. The topographic differentiation of the spatial distribution characteristics of water conservation was obvious. Because the geodetector can detect the global driving force, it can also detect the local driving force in different regions. To explore the influence of various driving factors on the spatial heterogeneity of water conservation at a deeper lever, we referred to the results of Wang et al. (2019) to divide the study area into plateaus, mountains, and plains (Figure 1). The driving factors of different regions had different effects on water conservation (Table 9; Figure 6). Among them, the water conservation capacity of the entire region was affected most significantly by soil‐saturated hydraulic conductivity (27.141%); the water conservation capacity of the plateaus was affected primarily by precipitation (33.408%); the water conservation capacity of the mountain was affected primarily by the plant‐available water content (21.586%); and the water conservation capacity of the plains was affected primarily by precipitation (28.198%).

**Table 9:** The *q* Values of the Driving Factors for Water Conservation on the Whole Region, the Plateaus, the Mountains, and the Plains

	Precipitation (%)	Potential evapotranspiration (%)	Temperature (%)	Land use (%)	Maximum root burial depth of soil (%)	Plant‐available water content (%)	Soil‐saturated hydraulic conductivity (%)	Percentage slope (%)	GDP (%)	Population (%)
The whole region	18.722	9.885	12.583	3.750	5.332	12.642	27.141	2.485	1.534	1.423
Plateaus	33.408	25.132	20.841	3.571	25.492	20.967	23.098	2.546	0.255	0.433
Mountains	16.004	3.615	1.019	8.024	8.513	21.586	20.548	5.388	5.468	3.144
Plains	28.198	11.622	23.678	4.930	7.564	11.136	26.969	0.056	7.057	9.857

The *q* values of precipitation, potential evapotranspiration, temperature, the maximum buried depth of soil roots, plant‐available water content, soil‐saturated hydraulic conductivity, percentage slope, GDP, and population are shown on the whole region, the plateaus, the mountains, and the plains (Table 9).

The *q* values of the driving factors for water conservation on the whole region, the plateaus, the mountains, and the plains are shown in Figure 6.

### 5.3 Spatial and Temporal Heterogeneity Analysis of Water Conservation

In terms of space, water conservation was affected mainly by soil‐saturated hydraulic conductivity (27.141%) and precipitation (18.722%). Soil permeability is an important index of evaluating water source conservation function. Generally speaking, the stronger the water infiltration capacity is, the stronger the water conservation capacity is. The soil‐saturated hydraulic conductivity in Yanshan, the Taihang Mountains, and the eastern coastal areas of the study area was strong (Figure 2f), so its water conservation capacity also was strong. The amount of precipitation depended on the intensity and duration of precipitation. The more precipitation there was, the stronger the water conservation capacity was. The precipitation in the mountainous area was larger than that in the plains, so its water conservation capacity was stronger. The water conservation capacity of mountains, plateaus, and plains was affected mainly by the soil‐saturated hydraulic conductivity (27.141%), plant‐available water content (21.586%), precipitation (33.408%), and precipitation (28.198%). Due to the difference of precipitation distribution in different topographic areas, the interpretation of precipitation on plateau and plain is not consistent. The vegetation coverage of the plateau and the plains was relatively low, so it was greatly affected by precipitation. Plant‐available water content was the proportion of water supply for plant growth in a soil layer. The higher the vegetation coverage was, the greater the water supply for plant growth, which meant that the water conservation capacity of this area was strong. The land use types in mountainous areas included mostly forestland, with relatively high vegetation coverage.

In terms of time, water conservation was mainly affected by climate and land use (Lang et al., 2017). Zeng and Li (2019) found that the key influencing factors impacting the water conservation included precipitation, evapotranspiration, and land use from 2005 to 2050 in the Weihe River. Han and Dong (2017) found that water supply was overwhelmingly driven by the reference evapotranspiration and annual average precipitation, while the plant evapotranspiration coefficients for each land use type had a relatively small effect since 1990s in Guizhou Province. On this basis, the authors mainly found that the combined effect of precipitation, potential evapotranspiration, and land use on water conservation was larger than any single factor of three.

**Figure 7:**
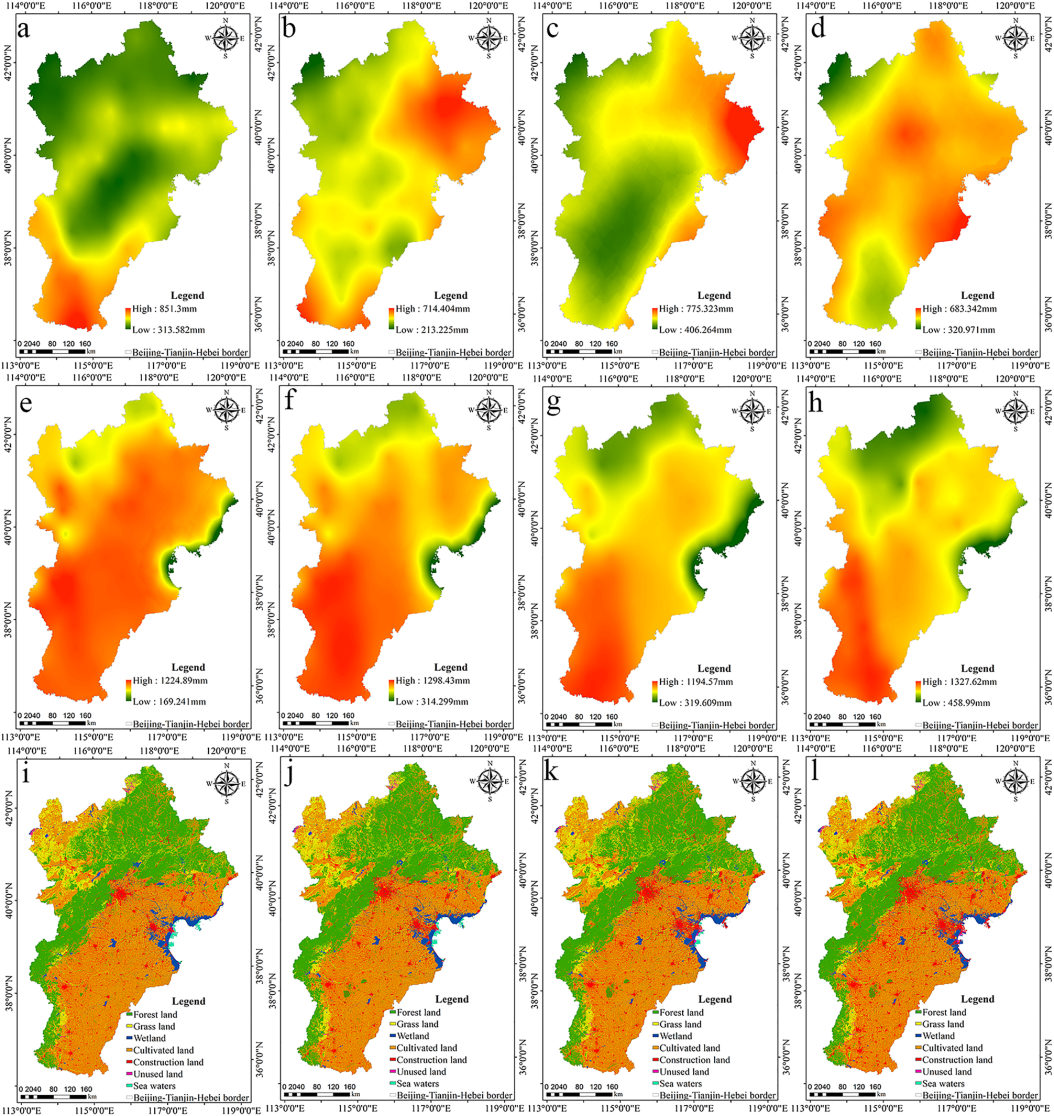
Spatial patterns of time driving factors: (a) precipitation in 2000, (b) precipitation in 2005, (c) precipitation in 2010, (d) precipitation in 2015, (e) potential evapotranspiration in 2000, (f) potential evapotranspiration in 2005, (g) potential evapotranspiration in 2010, (h) potential evapotranspiration in 2015, (i) land use in 2000, (j) land use in 2005, (k) land use in 2010, and (l) land use in 2015.

Water conservation capacity was sensitive to the change in precipitation, potential evapotranspiration, and land use in the entire region. The variation of precipitation was relatively large. On the whole, the precipitation showed an increasing trend. The regions with high precipitation were concentrated in a small part of the southern plain and the eastern coastal areas (Figures 7a–7d). Compared with precipitation, potential evapotranspiration showed a fluctuating trend. Potential evapotranspiration was the amount of water that may have been lost through soil evaporation and plant evapotranspiration when water was sufficient. In 2000–2015, the potential evaporation of Beijing‐Tianjin‐Hebei urban agglomeration remained unchanged as a whole, with slight differences in some areas. This showed spatial distribution characteristics of a gradual increase from north to south (Figures 7e–7h). The forestland in the study area was mainly distributed in the Yanshan and Taihang Mountains, and the grassland was mainly distributed in the northwest plateau area. The forestland had high vegetation coverage, strong rainfall interception capacity, and relatively high water conservation capacity. Cultivated land and artificial surfaces were distributed primarily in the central and southern plain areas. Compared with forestland, grassland, cultivated land, and artificial surfaces had less vegetation coverage, less rainfall interception capacity, and lower water conservation capacity. The distribution area of wetland, unused land, and sea area was relatively small, mainly in the eastern region. The areas with strong water conservation capacity in the study area were distributed mainly in forestland and eastern urban areas (Figures 7i–7l), mainly because of the high vegetation coverage, strong rainfall capacity, and strong soil‐saturated water diversion capacity.

It is helpful to develop reasonable and effective programs for water management by revealing factors to control water conservation. The urban water conservation capacity in the Yanshan and Taihang Mountains and the eastern coastal areas was relatively large, which primarily included Beijing, Qinhuangdao, Chengde, Zhangjiakou, Tangshan, and Handan. We should strengthen the protection of water sources and strengthen the protection and restoration of ecological systems in these extremely important water conservation areas. For areas with relatively poor water conservation capacity in the central and southern plains, it will be necessary not only to control environmental pollution but also to protect the water quality of the basin. In addition, we should increase vegetation coverage in these areas by returning farmland to forests.

The assessment of the spatial heterogeneity analysis of water conservation capacity presented some limitations. In terms of space, because the geodetector model was used to handle categorical variables, continuous data often must be discretized before modeling, which may result in information loss. In terms of time, due to the lack of relevant data, the authors mainly explored the interactive characteristics of precipitation, potential evapotranspiration, and land use on water conservation. In the next step of the study, more factors will be considered comprehensively.

The spatial patterns of precipitation, potential evapotranspiration, and land use from 2000 to 2015 are shown in Figure 7.

## 6 Conclusions

The relationship between water conservation and its driving factors holds great significance for regulating regional water resources. The authors offered an objective framework for revealing factors to control water conservation and for identifying the relationship in the elements driving the element set of water conservation. In the study, the authors used the InVEST model to calculate water yield from 2000 to 2015 and compared water resources bulletin data for model verification. The authors calculated water conservation capacity using the topographic index, soil‐saturated hydraulic conductivity, and flow coefficients. On this basis, the authors used a new method based on a geodetector and spatial elastic coefficient trajectory model to explore the spatial and temporal interaction characteristics between driving factors and water conservation. The spatial driving factors affecting water conservation included precipitation, potential evapotranspiration, temperature, land use, maximum root burial depth of soil, plant‐available water content, percentage slope, GDP, and population. The authors selected precipitation, potential evapotranspiration, and land use as the time‐driven factors. The results are as follows.

1. The strong water conservation capacity was reflected primarily in the Yanshan and Taihang Mountains and the eastern coastal areas.
2. The driving factors of different regions had different effects on water conservation. Among them, the water conservation capacity of the whole region, mountain, plateaus, and plains was affected mainly by the soil‐saturated hydraulic conductivity, plant‐available water content, precipitation, and precipitation.
3. Water conservation capacity was sensitive to the change of the precipitation, potential evapotranspiration, and land use in the entire region. Each driving factor exhibited a clearly interactive influence on the spatial distribution of water conservation in terms of space and time.

## Conflict of Interest

The authors declare no conflict of interest relevant to this study. The founding sponsors had no role in the design of the study; in the collection, analyses, or interpretation of data; in the writing of the manuscript; and in the decision to publish the results.

## Data Availability

All data sets used in this work are derived from sources that are freely and publicly available, as referenced in the text. We confirm that all additional data used in this paper can be found in figshare repository (https://figshare.com/s/1ac7c5d81bcc496d13ff).
